# Which prognostic marker is responsible for the clinical heterogeneity in CLL with 13q deletion?

**DOI:** 10.1186/s13039-020-00522-1

**Published:** 2021-01-06

**Authors:** Beyhan Durak Aras, Sevgi Isik, Hava Uskudar Teke, Abdulvahap Aslan, Filiz Yavasoglu, Zafer Gulbas, Fatih Demirkan, Hulya Ozen, Oguz Cilingir, Nur Sena Inci, Gulcin Gunden, Tuba Bulduk, Ebru Erzurumluoglu Gokalp, Sinem Kocagil, Sevilhan Artan, Olga Meltem Akay

**Affiliations:** 1 Department of Medical Genetics, Faculty of Medicine, University of Eskisehir Osmangazi, Professor Dr. Nabi Avcı Street, No. 4, Eskisehir, Buyukdere 26040 Turkey; 2 Department of Hematology, Faculty of Medicine, University of Eskisehir Osmangazi, Eskisehir, Turkey; 3 Department of Hematology, Private Umit Hospital, Eskisehir, Turkey; 4 Department of Hematology, Faculty of Medicine, University of Afyonkarahisar Health Sciences, Afyon, Turkey; 5 Department of Hematology, Anadolu Medical Center, Kocaeli, Turkey; 6 Department of Oncology, Faculty of Medicine, University of Dokuz Eylul, Izmir, Turkey; 7 Department of Biostatistics, Faculty of Medicine, University of Eskisehir Osmangazi, Eskisehir, Turkey; 8 Department of Hematology, Faculty of Medicine, University of Koc, Istanbul, Turkey

**Keywords:** B-CLL, FISH, Prognostic marker, *RB1* deletions, 13q deletions

## Abstract

**Background:**

Deletion of 13q14 [del(13q)] is the most common cytogenetic change (50%) in chronic lymphoblastic leukemia (CLL), and it is a good prognostic factor if it is detected as a sole aberration by FISH. However, it is observed the clinical course of CLL cases with del(13q) are quite heterogeneous and the responsible for this clinical heterogeneity has not been established yet. Some investigators suggest type II deletion (include *RB1* gene) is associated with more aggressive clinical course. Also, it is suggested that the deletion burden and the deletion type have a prognostic effect. In this study, we aimed to investigate the effect of *RB1* gene deletion, deletion burden and deletion type on overall survival (OS), disease stage and time to first treatment (TTFT) in patients with isolated del(3q). Sixty eight cases, detected isolated del(13q) were included in the study. Also, *RB1* deletion was analyzed from peripheral blood of them using FISH.

**Results:**

*RB1* deletion was detected in 41% of patients, but there was no statistically significant difference between *RB1* deletion and TTFT, stage and OS (*p* > 0.05). At same time, statistically significant difference was detected between high del(13q) (> 80%) and TTFT (*p* < 0.05).

**Conclusion:**

The statistical analysis of our data regarding to the association between *RB1* deletion and deletion type, TTFT, disease stage, and OS has not confirmed type II deletion or biallelic deletion cause poor prognosis. However, our data supports the deletion burden has a prognostic effect. More studies are needed to elucidate the cause of the clinical heterogeneity of CLL cases with del(13q).

## Background

Chronic lymphocytic leukemia (CLL) is a disease characterized by the increase of mature neoplastic B lymphocytes in lymphocytic tissues such as peripheral blood, bone marrow, lymph node, spleen and liver. One-third of the patients need treatment at the time of diagnosis, one-third of them progress and develop a need for treatment over the time, and one-third never need treatment. In addition to the Rai and Binet staging systems, there are many studies aimed at determining the prognosis in CLL. There are some independent parameters that have been shown to be important in terms of prognosis other than staging; lymphocyte count, bone marrow involvement level, atypical lymphocyte ratio and lymphocyte count doubling time (LDT), LDH, beta-2 microglobulin, thymidine kinase, sCD23 and sCD44, cytogenetic changes, IGH mutation status, CD38 expression rate, ZAP70 expression [1–3]. Although there is no single specific cytogenetic anomaly in CLL, the most common anomalies are 13q14 deletion (50%), 11q22–23 deletion (17–20%), trisomy 12 (15%) and 17p13 deletion. While deletion 13q14 correlates with long-term survival, trisomy 12 is associated with intermediate prognosis, the deletions 11q and 17p cause poor prognosis [2, 4].

Deletion of 13q14 [del(13q)] is the most common cytogenetic change (50%) CLL and it is a good prognostic factor if it is detected as a sole aberration by FISH. However, it is observed that the clinical course of CLL cases with del(13q) are quite heterogeneous and the reason for this clinical heterogeneity has not been established yet. Some investigators suggest type II deletion (include *RB1* gene) is associated with more aggressive clinical course. Also, it is suggested that the deletion burden and the deletion type (being homozygous and/or heterozygous) have a prognostic effect.

The size of chromosome 13 long arm deletions vary widely [5, 6]. In recent studies, 13q deletions were divided into two groups based on the size of deletion; Type 1 deletions contain the miR15a/16-1 deletions but not the *The retinoblastoma (RB1)* gene. Type 2 deletions, which are reported to be associated with more aggressive clinics, are defined as 13q deletions in which the *RB1* gene is also deleted [7, 8]. Some of the studies argue that the more the deletion burden increases, the more aggressive the disease progresses [9–11]. In addition, studies have reported that monoallelic 13q deletions are observed in the early stage and are associated with larger deletions, but biallelic deletions are associated with more aggressive clinical trials [6, 12].

The reason for the clinical heterogeneity observed in isolated del(13q) cases has not been clarified yet. In this study, we aimed to investigate the effect of *RB1* gene deletion, deletion burden and deletion type on overall survival (OS), disease stage and time to first treatment (TTFT) in patients with isolated del(3q). Sixty eight cases, where isolated del(13q) was detected,were included in the study. Also, *RB1* deletion was analyzed from peripheral blood of the cases using FISH.

## Results

Sixty eight CLL patients with isolated del(13q) detected by CLL FISH panel (*ATM, TP53*, 13q14 and trisomy 12) between 2006 and 2019 were included in our study, in which we aimed to investigate the cause of the clinical heterogeneity of CLL cases with del(13q).

At the time of enrollment, 46 patients (70.58%) were at RAI stage 0, I and II, 14 (20.58%) at stage III and stage IV. Rai stage status could not be determined in eight (11.76%) patients. While analysis 41 of the cases, whose mean (median) TTFT and OS durations were 28 and 52 months, had no treatment, 23 cases were receiving treatment but treatment information of 4 cases could not be reached. While the rate of 13q deletion was below 80% in 77% (52/68) of the cases, the rate of 13q deletion was found to be above 80% in 23% (16/68). In addition, deletion burden is included in Table 1 according to the cut off values of 60% and 70% in our case numbers.

**Table 1:** Clinical data and deletion 13q pattern of 68 CLL cases with isolated del 13q

	Frequency	Percent
Gender		
Female	28	41
Male	40	59
RAI stage		
Low (0, 1, 2)	46	70
High (4, 5)	14	20
Treatment		
Positive	23	33
Negative	41	60
Beta2 microglobulin		
< 3.5	18	55
> 3.5	15	45
*RB1* deletion		
Positive	27	40
Negative	41	60
13q deletion rate		
< 80%	52	77
> 80%	16	23
< 70%	46	68
> 70%	22	32
< 60%	40	59
> 60%	28	41
Deletion type		
Monoallelic	58	96
Biallelic	2	4

When we analysed the deletion type, a biallelic deletion 13q was detected in 6 cases. As a result of the FISH study, with the probe targeting the *RB1* region, *RB1* gene deletion was detected in 27 (40%) of the cases (Table 1).

**Fig. 1:**
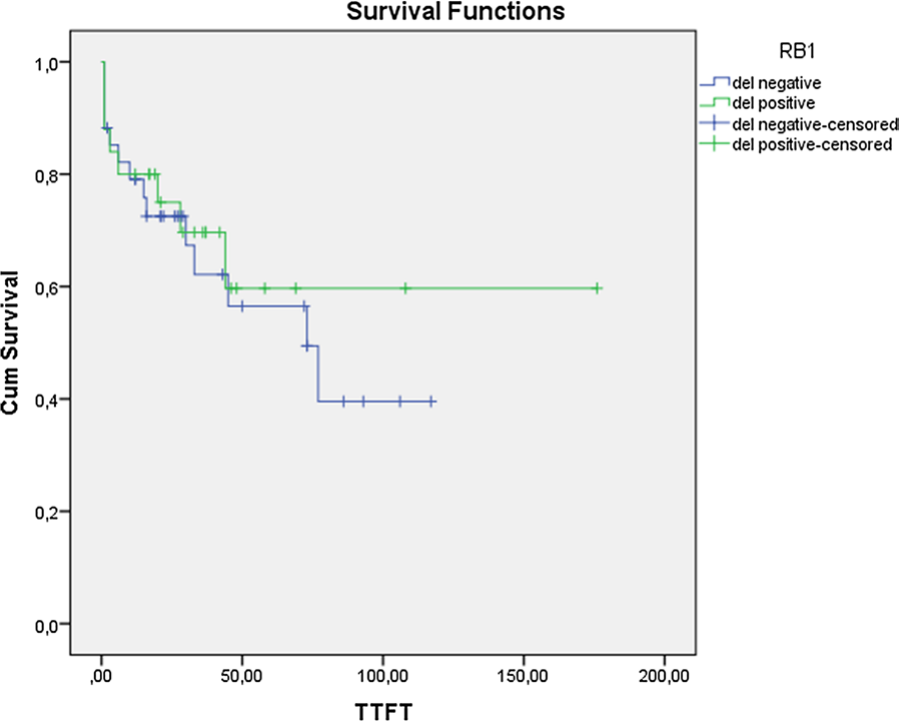
Relationship between *RB1* gene deletion (del) and TTFT (time to first treatment)

As a result of the statistical analysis, no statistically significant relationship was found between the *RB1* gene deletion positive and negative cases in terms of time to start treatment, disease stage, treatment status, and β2 microglobulin levels (*p* > 0.05) (Fig. 1). In addition, there was no statistically significant relationship between *RB1* gene deletion and the percentage of 13q deletion and the type of 13q deletion. The data of the cases are included in Table 2.

**Table 2:** Relationship between *RB1* gene deletion and disease stage, treatment status, β2 microglobulin level, 13q deletion rate and type

	*RB1* gen deletion		Total	*p* value
	Negative n (%)	Positive n (%)		
RAI stage				
0, 1, 2	25 (54)	21 (46)	46	*p* > 0.05
3, 4	9 (64)	5 (36)	14	
Total	34	26	60	
Treatment				
Positive	15 (62)	8 (35)	23	*p* > 0.05
Negative	24 (58)	17 (42)	41	
Total	39	25	64	
β2 microglobuline				
< 3.5 mg/dl	11 (61)	7 (39)	18	*p* > 0.05
> 3.5 mg/dl	9 (60)	6 (40)	15	
Total	20	13	33	
13q deletion rate				
< 80%	33 (63)	19 (37)	52	*p* > 0.05
> 80%	8 (50)	8 (50)	16	
Total	41 (60)	27 (40)	68	
< 70%	23 (60,5)	15 (39,5)	38	*p* > 0.05
> 70%	8 (42)	11 (58)	19	
Total	31 (54)	26 (46)	57	
< 60%	19 (59)	13 (41)	32	*p* > 0.05
> 60%	12 (48)	13 (52)	25	
Total	31 (54)	26 (46)	57	
Deletion type				
Monoallel	37 (59)	26 (41)	63	*p* > 0.05
Biallel	4 (80)	1 (20)	5	
Total	41	27	68	

**Fig. 2:**
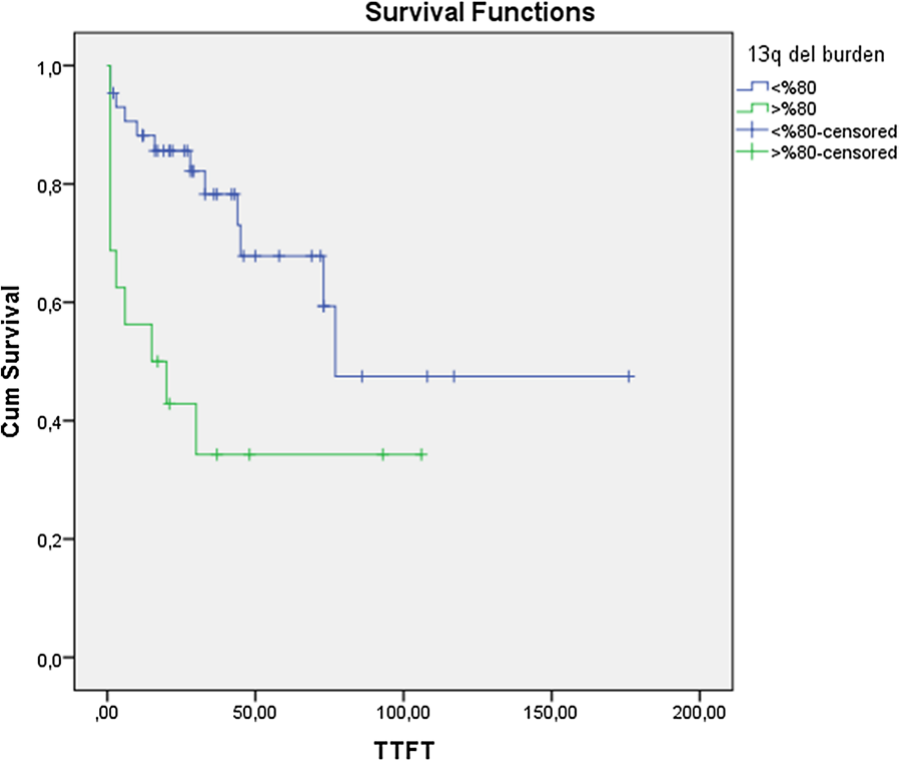
Relationship between deletion 13q (13q del) burden and TTFT (time to first treatment)

When we grouped our cases by determining 3 different cut-offs for 13q deletion rates (60%, 70% and 80%), it was found that the time to initiate treatment was statistically significantly shorter in the cases with deletion rate over 80% compared to the cases with less than 80% (*p* < 0.05) (Fig. 2).

As a result of the analysis, there was no statistically significant relationship between the 13q deletion rate and the RAI stages and β2 microglobulin levels (*p* > 0.05), but a statistically significant relationship was found between the cases with a 13q deletion rate above 80% and the patients receiving treatment (*p* < 0.05). The findings of the cases regarding the 13q deletion percentage rates are given in Table 3.

**Table 3:** Relationship between 13q deletion rates and treatment status, β2 microglobulin levels and RAI stages

	13q deletion rate											
	< 80% n (%)	> 80% n (%)	Total	*p* value	70% n (%)	> 70% n (%)	Total	*p* value	< 60% n (%)	> 60% n (%)	Total	*p* value
Treatment												
Positive	13 (56)	10 (44)	23	***p* < 0.05**	14 (61)	9 (39)	23	*p* > 0.05	11 (48)	12 (52)	23	*p* > 0.05
Negative	35 (85)	6 (15)	41		29 (71)	12 (29)	41		25 (61)	16 (39)	41	
Total	48 (75)	16 (25)	64		43 (67)	21 (33)	64		36 (56)	28 (44)	64	
β2 microglobuline level												
<3.5 mg/dl	14 (78)	4 (22)	18	*p* > 0.05	14 (78)	4 (22)	18	*p* > 0.05	12 (67)	6 (33)	18	*p* > 0.05
>3.5 mg/dl	12 (80)	3 (20)	15		11 (73)	4 (27)	15		10 (67)	5 (33)	15	
Total	26 (79)	7 (21)	33		25 (76)	8 (24)	33		22 (67)	11 (33)	33	
RAI stage												
0, 1, 2	37 (80)	9 (20)	46	*p* > 0.05	31 (67)	15 (33)	46	*p* > 0.05	28 (61)	18 (39)	46	*p* > 0.05
3, 4	8 (57)	6 (43)	14		9 (64)	5 (36)	14		6 (43)	8 (57)	14	
Total	45 (75)	15 (25)	60		40 (67)	20 (33)	60		34 (57)	26 (43)	60	

Bold value indicate statistically significant *p* < 0.05

Furthermore, as a result of the statistical analysis, no relationship was found between the 13q deletion type (mono and/or biallelic) and the disease stage (*p* = 0.051) (Table 4).

**Table 4:** Relationship between deletion 13q type and disease stage

	13q Deletion type			*p* value
	Monoallelic	Biallelic	Total	
RAI stage				
0, 1, 2	46 (100)	0 (0)	46	**0.051**
3, 4	12 (86)	2 (14)	14	
Total	58 (97)	2 (3)	60	

Bold value indicate statistically significant *p* < 0.05

## Discussıon

Chronic lymphocytic leukemia is a genetically heterogeneous disease, and has been defined as a genetic anomaly having recurrent prognostic effects through the course of time. While some of the del(13q) cases, which are most frequently observed and associated with good prognosis when observed in isolation, have been followed without treatment for years, it is revelaed that some of them have a much more aggressive clinic and resulted in death even if the treatment started at the time of diagnosis [2, 11]. In routine, the FISH method is used for the detection of 13q14 deletions and the deletion load can be calculated with FISH. In a study, comparing the efficiency of MLPA with cytogenetic and FISH, it was shown that patients with del(13)(q14.3q14.3) > 70% had an unfavorable prognosis and it was stated that FISH studies were also necessary for CLL patients with del(13q) in MLPA [13]. Hernandez et al. found in their study that isolated del(13q) CLL cases with a deletion load of more than 80% had a statistically significantly shorter TTFT and OS than those with a deletion burden of less than 80%, and showed 13q deletion burden had an effect on the prognosis [10]. In another study, a statistically significant difference was found in terms of TTFT between CLL cases with deletion burden above 65% and below 65%, but no difference was observed in terms of OS [14]. In a study conducted in 2013, it was reported that isolated del(13q) detected above 90% cannot be associated with good prognosis and are an independent prognostic marker [15]. In another study, statistically significantly shorter TTFT was found in cases with more than 70% deletion rate [16]. Miao et al. accepted the cases with more than 80% 13q deletions as high deletions and reported that high deletion rate was associated with short TTFT, Binet A and B IGHV mutation positive and caused poor prognosis [11]. Working with a much larger number of cases, Dal Bo et al. determined the cut off value as 70% in their study with 342 isolated del(13q) CLL cases, and found a relationship between deletion load over 70% and short TTFT and OS [9]. In another study aiming to reveal the cause of clinical heterogeneity in cases with Del(13q), significant differences were found in the expressions of some genes and some microRNAs that affect apoptosis and proliferation in cases with a deletion rate above 80%. It has also been stated that gene expression patterns of cases with high del(13q) could be similar to those of deletion 11q and 17p cases [17]. When we reviewed the literature data, we observed that the cut off values determined for deletion burden varied (65.5–90%). In our study, we determined three different cut off values as 60, 70 and 80%. When we investigated whether there was a difference between the groups below and above the cut-off values in terms of TTFT and disease stage, it was found that the TTFTs of the cases with del(13q) above 80% cut-off values were significantly shorter and a relationship between deletion rate above 80% and receiving treatment was shown. In line with all these data, genetic analysis results in CLL cases should be evaluated not only with del(13q), but also by considering the deletion burden. The results of our study suggest that it is important to evaluate the del(13q) burden as a prognostic marker.

*RB1* gene is a tumor suppressor gene located in the 13q14 band on the 13th chromosome. Retinoblastoma protein (pRB) is a tumor suppressor protein that controls the G1/S checkpoint of the cell cycle. Loss or mutation in the *RB1* tumor suppressor gene; contributes to the development of many cancers, including cancers such as breast, bone, lung and bladder [18]. In a study conducted with CLL patients in 2008, type 2 del(13q) was found to be associated with high Rai stage and aggressive clinic [6]. In 2011, Parker et al. found *RB1* deletions to be associated with disease progression [8]. In another study, while *RB1* deletions were found to be associated with short OS, no relationship was found with TTFT [19]. In later microarray studies, type 2 deletions were found to be associated with short TTFT [10, 20]. However, there are some studies stating otherwise, where *RB1* deletions to be associated with TTFT and OS were not found [21, 22]. As a result of the data obtained in our study, no clinical difference was found regarding *RB1* gene deletion positive and negative cases in terms of TTFT, disease stage, and whether or not they received treatment. Therefore, our study support that *RB1* gene deletions have no prognostic effect.

However, as we mentioned above, Dal Bo et al. who found that 13q deletion load above 70% was associated with short TTFT and OS, stated that deletion size and deletion load should be evaluated together. In their study, it was reported that if del(13q) was below 70% and type 1, it had a good prognostic effect, if the deletion burden was above 70% (type 1 or 2) or less than 70% but type 2 deletion was associated with short TTFT [9]. In addition, in the studies of Huang et al. only *RB1* gene deletion was evaluated and no significant relationship was found between *RB1* gene deletion and TTFT and OS. However, the deletion load being higher than 60% and the presence of *RB1* gene deletion were found to be associated with short TTFT [23]. In our study, when the deletion burden and *RB1* gene deletion were evaluated together, it was found that *RB1* gene deletions had no prognostic effect.

Ouillette et al. detected the frameshift mutation of *RB1* in 2 of 53 cases with type II 13q deletions [19]. It is also known that copy neutral loss of heterozygosity (Uniparental disomy) occurs in the 13q14 chromosome region [21]. *RB1* null (made by mutations, epigenetic mechanisms etc.) may cause clinical heterogeneity. The deficiency of our study is that *RB1* mutations were not examined. We are planning to perform mutation analyzes including the *RB1* gene and microarray analyzes in the continuation of the study in the future.

There are conflicting data in the literature about the effect of deletion type on the clinical heterogeneity of cases with isolated del(13q). A study conducted in 2008 suggested that biallelic 13q deletions were associated with more aggressive clinical presentation (high disease stage, high lactate dehydrpgenase level, short TTFT) and that biallelic deletions may have developed from monoallelic 13q deletions by clonal evolution [24]. In 2012, Orlandi et al. found that biallelic deletions were found to be associated with short TTFT and binet C phase. However, they stated that the deletion type cannot be defined as an independent prognostic factor, since biallelic dell3q cases also had a high deletion burden [16]. In another study conducted with 323 isolated del(13q), it was reported that the deletion type had no effect on TTFT and OS [14]. In another study investigating the prognostic effect of deletion type in 176 isolated del(13q) CLL patients, no difference was observed between the biallelic and monoallelic del(13q) groups in terms of TTFT and disease stage [25]. As a result of our study, it was observed that the biallelic deletion type did not have an effect on TTFT, β2 microglobulin level and disease stage, whether treatment was received or not. However, as a result of the statistical analysis investigating the relationship between deletion type and disease stage, as the p value was 0.05, it was thought that a significant relationship could be found between biallelic 13q deletion and high RAI stage by increasing the number of cases (Table 4). In our study, the number of biallelic del(13q) cases were 6 (3%), and they all showed a mosaic biallelic deletion pattern with monoallelic clones. It has been found in the literature that the biallelic deletion rates (mosaic and/or nonmosaic) vary between 5.5 and 39% [14, 16, 24, 25]. In addition, unfortunately, the disease stage information of the eight cases was not found in our study.

In conclusion, based on the data of our study, it was concluded that high deletion burden was associated with short TTFT in del(13q) CLL cases, but *RB1* deletion and deletion type did not have a prognostic effect. Considering the literature data, it is observed that there are conflicting data among the studies investigating the cause of the clinical heterogeneity of CLL cases with del(13q). It is concluded that further studies with larger case series are needed to clarify the cause of this heterogeneity.

## Materıal and methods

To investigate the cause of clinical heterogeneity in CLL patients with isolated deletion 13q, sixty-eight CLL cases with isolated 13q deletion (68 men, 28 women; mean age 675,970 ± 9.44 years) were enrolled. These patients were previously examined by FISH test for *ATM, TP53*, 13q14 deletions and trisomy 12 between 2006 and 2019 and were found to be positive for 13q14 deletion and negative for other anomalies. The diagnosis of CLL was based on the finding of peripheral lymphocytosis of 5 × 10^9^/L presenting the CD19, CD5, CD23 phenotype and kappa or lambda monoclonality.

This study was conducted according to the guidelines that were decelerated in the Declaration of Helsinki, and approved by the Clinical Practice Ethics Committee. Each individual provided signed consent form.

### Sample preperation

Peripheral blood (PB) samples from 68 CLL cases with isolated 13q deletion were directly cultured for in RPMI-1640 medium without mitogen stimulation. After the planting, standard harvest procedure (0.075 M KCl treatment and fixation with Carnoy’s fixative) was performed immediately.

### Slide pretreatment and FISH

Slides were prepared by dropping cell suspensions onto frozen microscopic slides and then left to dry overnight at room temperature before use.

In the FISH analysis probe; *RB1* Deletion Probe (Cytocell); for *RB1* gene region and 13qter as a control for chromosome 13 were used.

FISH was performed according to manufacturer’s specifications. Probes were denatured at 73 °C ± 1 °C for 5 min and then they were applied immediately to the previously determined regions of the slides. Following overnight hybridization at 37 °C, post hybridization washes were performed and air-dried in darkness. The slides were counterstained by using DAPI (4′-6′-diamidine-2-phenylindole) and were stored at − 20 °C in the dark.

Also, in the previous FISH study, D13S319 Plus Deletion Probe (Cytocell) (part of *DLEU1* gene and *DLEU2* gene and the D13S319 and D13S272 markers, *LAMP1* gene and 13q subtelomere) targeting the 13q14 region was used.

### Microscopy

Slides were analyzed with Olympus BX61 fluorescence microscope and images captured with a CCD camera using image analysis system (CytoVision Leica Biosystems). At least 200 nuclei using areas of the slides on which the cells were spread were analyzed. The cut-off points for positive values were determined for each probe from five bone-marrow samples collected from individuals with iron deficiency anemia.

The cut-off values for each probe are as following: D13S319 Plus Deletion Probe 8%, *RB1* Deletion Probe 10%.

### Statistical analysis

*RB1* deletion, deletion type and deletion burden were compared with clinical features using Fisher’s exact test and Kruskal–Wallis rest. TTFT was defined as the time from diagnosis to date of initial treatment or last follow up. The association between TTFT and *RB1* deletion, deletion burden and deletion type were evaluated by Kaplan–Meier method. For these analyses, Statistical Packege for the Social Sciences version 25 was used. For all tests, *p* < 0.05 was regarded statistically significant.

## Data Availability

All data generated or analysed during this study are included in this published article and its supplementary information files.

## Abbreviations

CLL: Chronic lymphocytic leukemia
FISH: Fluorescence in situ hybridization
Del: Deletion
TTFT: Time to first treatment
OS: Overall survival

## References

[1] Bosch F, Dalla-Favera R (2019). Chronic lymphocytic leukaemia: from genetics to treatment. Nat Rev Clin Oncol.

[2] Döhner H, Stilgenbauer S, Benner A, Leupolt E, Kröber A, Bullinger L (2000). Genomic aberrations and survival in chronic lymphocytic leukemia. N Engl J Med.

[3] O’Reilly A, Murphy J, Rawe S, Garvey M (2018). Chronic lymphocytic leukemia: a review of front-line treatment options, with a focus on elderly CLL patients. Clin Lymphoma Myeloma Leuk.

[4] Durak B, Akay OM, Aslan V, Ozdemir M, Sahin F, Artan S (2009). Prognostic impact of chromosome alterations detected by FISH in Turkish patients with B-cell chronic lymphocytic leukemia. Cancer Genet Cytogenet.

[5] Mosca L, Fabris S, Lionetti M, Todoerti K, Agnelli L, Morabito F (2010). Integrative genomics analyses reveal molecularly distinct subgroups of B-cell chronic lymphocytic leukemia patients with 13q14 deletion. Clin Cancer Res.

[6] Ouillette P, Erba H, Kujawski L, Kaminski M, Shedden K, Malek SN (2008). Integrated genomic profiling of chronic lymphocytic leukemia identifies subtypes of deletion 13q14. Cancer Res.

[7] Hanlon K, Ellard S, Rudin CE, Thorne S, Davies T, Harries LW (2009). Evaluation of 13q14 status in patients with chronic lymphocytic leukemia using single nucleotide polymorphism-based techniques. J Mol Diagn.

[8] Parker H, Rose-Zerilli MJ, Parker A, Chaplin T, Wade R, Gardiner A (2011). 13q deletion anatomy and disease progression in patients with chronic lymphocytic leukemia. Leukemia.

[9] Dal Bo M, Rossi FM, Rossi D, Deambrogi C, Bertoni F, Del Giudice I (2011). 13q14 deletion size and number of deleted cells both influence prognosis in chronic lymphocytic leukemia. Genes Chromosomes Cancer.

[10] Hernández JA, Rodríguez AE, González M, Benito R, Fontanillo C, Sandoval V (2009). A high number of losses in 13q14 chromosome band is associated with a worse outcome and biological differences in patients with B-cell chronic lymphoid leukemia. Haematologica.

[11] Miao Y, Miao Y, Shi K, Sun Q, Zhao SS, Xia Y (2018). A higher percentage of cells with 13q deletion predicts worse outcome in Chinese patients with chronic lymphocytic leukemia carrying isolated 13q deletion. Ann Hematol.

[12] Shanafelt TD, Witzig TE, Fink SR, Jenkins RB, Paternoster SF, Smoley SA (2006). Prospective evaluation of clonal evolution during long-term follow-up of patients with untreated early-stage chronic lymphocytic leukemia. J Clin Oncol.

[13] Alhourani E, Rincic M, Othman MA, Pohle B, Schlie C, Glaser A (2014). Comprehensive chronic lymphocytic leukemia diagnostics by combined multiplex ligation dependent probe amplification (MLPA) and interphase fluorescence in situ hybridization (iFISH). Mol Cytogenet.

[14] Van Dyke DL, Shanafelt TD, Call TG, Zent CS, Smoley SA, Rabe KG (2010). A comprehensive evaluation of the prognostic significance of 13q deletions in patients with B-chronic lymphocytic leukaemia. Br J Haematol.

[15] Puiggros A, Delgado J, Rodriguez-Vicente A, Collado R, Aventín A, Luño E (2013). Biallelic losses of 13q do not confer a poorer outcome in chronic lymphocytic leukaemia: analysis of 627 patients with isolated 13q deletion. Br J Haematol.

[16] Orlandi EM, Bernasconi P, Pascutto C, Giardini I, Cavigliano PM, Boni M (2013). Chronic lymphocytic leukemia with del13q14 as the sole abnormality: dynamic prognostic estimate by interphase-FISH. Hematol Oncol.

[17] Rodríguez AE, Hernández J, Benito R, Gutiérrez NC, García JL, Hernández-Sánchez M (2012). Molecular characterization of chronic lymphocytic leukemia patients with a high number of losses in 13q14. PLoS ONE.

[18] Knudsen ES, Knudsen KE (2008). Tailoring to RB: tumour suppressor status and therapeutic response. Nat Rev Cancer.

[19] Ouillette P, Collins R, Shakhan S, Li J, Li C, Shedden K (2011). The prognostic significance of various 13q14 deletions in chronic lymphocytic leukemia. Clin Cancer Res.

[20] Mian M, Rinaldi A, Mensah AA, Rossi D, Ladetto M, Forconi F (2012). Del(13q143) length matters: an integrated analysis of genomic, fluorescence in situ hybridization and clinical data in 169 chronic lymphocytic leukaemia patients with 13q deletion alone or a normal karyotype. Hematol Oncol.

[21] Grygalewicz B, Woroniecka R, Rygier J, Borkowska K, Rzepecka I, Lukasik M (2016). Monoallelic and biallelic deletions of 13q14 in a group of CLL/SLL patients investigated by CGH Haematological Cancer and SNP array (8 × 60 K). Mol Cytogenet.

[22] Houldsworth J, Guttapalli A, Thodima V, Yan XJ, Mendiratta G, Zielonka T (2014). Genomic imbalance defines three prognostic groups for risk stratification of patients with chronic lymphocytic leukemia. Leuk Lymphoma.

[23] Huang SJ, Gillan TL, Gerrie AS, Hrynchak M, Karsan A, Ramadan K (2016). Influence of clone and deletion size on outcome in chronic lymphocytic leukemia patients with an isolated deletion 13q in a population-based analysis in British Columbia, Canada. Genes Chromosomes Cancer.

[24] Chena C, Avalos JS, Bezares RF, Arrossagaray G, Turdó K, Bistmans A (2008). Biallelic deletion 13q14.3 in patients with chronic lymphocytic leukemia: cytogenetic, FISH and clinical studies. Eur J Haematol.

[25] Garg R, Wierda W, Ferrajoli A, Abruzzo L, Pierce S, Lerner S (2012). The prognostic difference of monoallelic versus biallelic deletion of 13q in chronic lymphocytic leukemia. Cancer.

